# Balancing Efficiency and Access: Discouraging Emergency Department Boarding in a Global Budget System

**DOI:** 10.5811/westjem.2021.5.51889

**Published:** 2021-09-02

**Authors:** Benoit Stryckman, Diane Kuhn, Daniel B. Gingold, Kyle R. Fischer, J. David Gatz, Stephen M. Schenkel, Brian J. Browne

**Affiliations:** University of Maryland School of Medicine, Department of Emergency Medicine, Baltimore, Maryland

## Abstract

Reducing cost without sacrificing quality of patient care is an important yet challenging goal for healthcare professionals and policymakers alike. This challenge is at the forefront in the United States, where per capita healthcare costs are much higher than in similar countries around the world. The state of Maryland is unique in the hospital financing landscape due to its “capitation” payment system (also known as “global budget”), in which revenue for hospital-based services is set at the beginning of the year. Although Maryland’s system has yielded many benefits, including reduced Medicare spending, it also has had unintentional adverse consequences. These consequences, such as increased emergency department boarding and ambulance diversion, constrain Maryland hospitals’ ability to fulfill their role as emergency care providers and act as a safety net for vulnerable patient populations. In this article, we suggest policy remedies to mitigate the unintended consequences of Maryland’s model that should also prove instructive for a variety of emerging alternative payment mechanisms.

## Maryland’s Unique Hospital Financing System that Caps Revenue, Incentivizing Cost Savings

For over 40 years, Maryland’s hospitals have occupied a unique niche in the US healthcare financing landscape. Under a Medicare waiver in 1977, Maryland began setting the prices hospitals can charge for services, known as an all-payer rate-setting system, with all third parties paying the same rate. In 2014 the Centers for Medicare and Medicaid Services (CMS) and the state of Maryland announced a new model that limits per capita expenditures for hospital services, with the aim to incentivize more deliberate spending decisions, to use hospital resources more efficiently, and improve quality of care. Maryland converted from a regulated fee-for-service model to a quasi-state-managed capitated payment system, ie, global budget, in which revenue for hospital-based services is set at the beginning of the year. In 2019 the state of Maryland and CMS agreed to reform the concept as the “Total Cost of Care Model.” This reform continues the global budgeting system while adding new programs to incentivize collaborations between hospital and non-hospital providers, as well as expands the role of primary care providers in prevention, chronic disease management, and reduction of unnecessary hospital utilization.^1^–^3^

In principle, the global budget is relatively simple; in practice, the financing mechanism is dynamic and requires careful monitoring by the state’s regulatory body, the Health Services Cost Review Commission (HSCRC).^4^ The HSCRC manages the global budget across a number of domains to ensure hospital revenue is distributed accurately, regularly adjusting for factors such as “changes in service levels, market share shifts, or shifts of services to unregulated settings.”^5^ In addition, payment based on a variety of quality metrics can increase or decrease hospital revenue. Managed at a state rather than a federal level, these quality metrics can be adjusted for local or state needs and may differ from metrics familiar to the rest of the country.^6^

Since implementation, Maryland’s model has slowed Medicare spending in the state’s hospitals compared to the rest of the nation.^7^ Maryland hospitals achieved this reduction through cost savings, shifting care to the outpatient setting, increasing hospital investment in care coordination, targeting potentially avoidable admissions, and minimizing wasteful use of resources.^4^,^8^ Maryland’s global budget system, which shifts financial risk from payers to hospitals, is designed to incentivize cost savings through efficient use of hospital resources. Unfortunately, inefficiencies may be difficult for hospital administrators to identify and address in practice. Instead, hospitals may respond to global budget incentives by taking an easier and more predictable approach: allocating fewer “capitated” funds to services with high cost-saving potential (such as 24/7 staffed inpatient beds). This is the opposite of the incentive structure under a traditional fee-for-service model, where an empty bed represents an opportunity for additional revenue. Absent compensatory policy guardrails, this response to global budget incentives can be expected to have downstream negative consequences on outpatient services with less cost-saving potential such as the emergency department (ED).

## Hospital Cost Savings May Unintentionally Contribute to Emergency Department Boarding and Ambulance Diversion

Well-intended interventions often come with unintended consequences after implementation in the real world. Hospital funding is no exception. While Maryland’s global budget model has helped slow the growth of healthcare costs, it has also impacted access to timely emergency care.

First, ED boarding causes reduced access to emergency services. “Boarding” occurs when patients admitted to the hospital wait in the ED until a staffed inpatient bed is available. The consequent crowding of the ED delays the evaluation of newly arriving patients. “Crowding” occurs when patients’ needs exceed available ED resources. Maryland has historically had high rates of ED boarding compared to other states (mean of 6 vs 5 hours in 2014).^9^ Still, these metrics worsened after the implementation of the capitation system.

**Figure 1:**
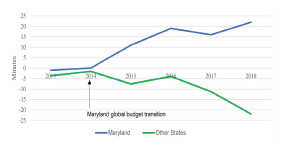
Cumulative absolute change in time from emergency department (ED) arrival to ED departure for admitted ED patients since 2013. Note. Emergency department boarding was 367 minutes in Maryland and 295 minutes in all other states, in 2012. Source: Hospital Compare.^9^

Between 2014–2018, the cumulative change in average time admitted patients boarded in the ED increased by an average of eight minutes annually in Maryland, whereas it decreased by an average of four minutes annually in other parts of the country (Figure 1). A recent study concluded that the global budget resulted in a statistically significant increase in ED boarding.^10^ This is unlikely to have been due to changes in utilization, as ED visits per 1000 residents decreased from 2012 to 2017 in Maryland.^11^ Another recent study indicated that the global budget led to modest declines in ED utilization the year after implementation in Maryland.^12^ Increased ED boarding in Maryland since 2014 coincides with the payment structure change and places the state on a divergent trajectory from other states, where boarding has been stable or decreasing over the same period.

Second, ED boarding affects not only patients already in the hospital, but those with emergencies outside the hospital as well. Hospitals place themselves on “diversion” status when the hospital and/or ED are overloaded. The emergency medical services (EMS) system may place a hospital on “re-route” status if ED crowding prevents ambulances from quickly unloading patients. These statuses signal EMS providers to take patients to another hospital, even if the initial hospital is closer or the patient has a previous care relationship there.

The Maryland Institute for Emergency Medical Services System (MIEMSS) has a tracking system that uses “yellow” alerts to indicate a facility is overwhelmed and unable to receive patients in urgent need of medical evaluation, and “red” alerts to indicate there are no available monitored beds in the hospital (including telemetry and critical care). While MIEMSS defines criteria for alerts, hospitals are responsible for placing themselves on and off alert, and not all hospitals follow the criteria in the same way.^13^ There is, therefore, variability in the use of these statuses when one compares hospital to hospital. However, hospital policies are unlikely to change very much across years. Total diversion hours are a good measure of state ED availability, and relative changes in diversion hours are likely a good proxy for trends in ED crowding. Re-route assignment, which is largely controlled by EMS and not by the hospitals, provides an alternative mechanism to measure ED crowding.

**Figure 2:**
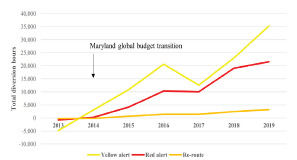
Cumulative absolute change in ambulance diversion time by diversion type in Maryland since 2013. Note. Diversion hours were yellow alert =17,377, red alert = 7648, and re-route = 1396 in 2012. Source: Maryland Institute for Emergency Medical Services Systems.^13^

The average total hours of yellow and red alert diversion status in Maryland rose by 23% and 32%, respectively, after the implementation of the global budget in 2014. Similarly, EMS-designated re-route diversion times have grown by 32% in the same period (Figure 2). Higher rates of diversion disrupt continuity of care and have been associated with delays in hospital arrival and increased mortality.^14^

## Emergency Department Boarding and Ambulance Diversion Have Significant Negative Consequences on Patient Care

The ED plays a critical function in the healthcare system. With 24/7 access to a hub of medical services, the ED is an entry point to the hospital for critically ill and injured patients and serves as the safety net for patients facing barriers to care in other parts of the system. Promptly transitioning patients from the ED to an inpatient setting allows new ED patients to be evaluated and admitted patients to have their care assumed by appropriate specialists and tertiary care teams. Delays in transitioning patients, as well as boarding admitted patients in the ED, result in increased ED crowding, itself a significant threat to patient safety and equity. This crowding in turn can lead to ambulance diversions and decreased access to emergency care.

An expanding body of evidence demonstrates the significant burden that ED boarding places on both individual patients—through delayed inpatient care and medical errors—and the hospital system through ambulance diversions. A 2018 systematic review of ED boarding found that nearly every one of the 102 reviewed studies observed worse quality of care for boarding patients,^15^ including delays in patient assessment and definitive treatment for conditions such as sepsis, pneumonia, myocardial infarction, and fractures.^16^–^18^ Medical errors are more common, and mortality rates are higher for patients admitted to the hospital when the ED is crowded.^19^ Crowding exacerbates health disparities by disproportionally impacting patient populations with barriers to care outside the ED, including patients who are poor, minorities, immigrants, and those insured by Medicare or Medicaid.^20^ Finally, boarding impairs an ED’s ability to respond to unexpected disasters that cause a large number of individuals to become ill or injured.

Just as the boarding patients themselves are negatively affected, so too are other patients in the ED awaiting workup and disposition. Although practices vary among hospitals and admitting services, it is common for the ED to retain some or most of the responsibility for patients who are admitted to an inpatient service but boarding in the ED:

- ED nurses monitor these patients’ clinical status and administer their medications.
- ED providers must remain aware of boarding patients’ clinical status and sign out their presence and needs at every shift change.
- In some cases, ED providers still place the patients’ orders and perform the necessary procedures for their care until the patient is physically transferred.
- ED staff receive calls from family members, lab technicians, and imaging specialists regarding boarding patients.

This responsibility impacts other ED patients as it places a burden on ED staff to care for both new patients as well as admitted patients, delaying evaluation and management for all ED patients.

Patients waiting to be seen may also choose to leave the ED without full evaluation and treatment. Not only is this an important quality metric for EDs, but patients who leave without being seen are at higher risk for adverse outcomes.^21^ Patient privacy, confidentiality, and satisfaction are also negatively affected.^18^

## Discussion and Future Directions

The Maryland global budget approach is designed to incentivize cost savings from efficient use of hospital resources with a focus on population health. While cost savings is an essential component of managing growth in health expenditures, action must be taken to ensure that hospitals’ cost-saving initiatives do not adversely affect access to emergency services and patient care.

Given the established relationship between a shortage of available staffed inpatient beds and ED boarding, it is necessary to examine how the cost-saving incentives of Maryland’s global budget system relate to hospital bed availability.^22^ The lack of a bed can be the result of either infrastructure or staffing limitations. Infrastructure limitations imply too few beds in the system and can only be resolved by limiting admissions or adding beds. Staffing limitations, on the other hand, often result from hospital administrators’ decision to balance bed availability with labor costs; this type of bed shortage is both more theoretically likely to result from the incentives of a global budget system and more amenable to rapid re-evaluation and correction. Of note, boarding should not be confused with deliberately keeping patients in outpatient observation status.

Both the HSCRC and MIEMSS have recognized ED boarding as a problem in Maryland. Since part of Maryland’s formula for hospital payment is based on individual hospital quality metrics, a 2017 Performance Measurement Work Group explored the addition of ED boarding metrics into Maryland’s Quality-Based Reimbursement (QBR) program. In 2017, the HSCRC proposed adding the Hospital Consumer Assessment of Healthcare Providers and Systems metric ED-2b (admit decision time to ED departure time for admitted patient) from the CMS to hospital reimbursement under the QBR program for rate year 2020. The MIEMSS has also written in favor of such metrics.^11^

Data for the ED-2b metric was collected as part of CMS’s Hospital Compare dataset. However, CMS removed the ED-2b metric in the process of revising measures from its hospital Inpatient Quality Reporting program “to focus measurement on the most critical quality issues with the least burden for clinicians and providers.”^23^ The HSCRC felt that if CMS wasn’t going to require collection of ED-2b, this change “necessitat[ed] its removal” from the Maryland QBR program as well. One reason for this sense of necessity was that the CMS Hospital Compare data was the source of reporting for Maryland’s QBR program, and without these data, HSCRC would need to establish its own ED data reporting infrastructure. Furthermore, HSCRC noted that in the short time ED-2b was included in Maryland’s QBR program, little progress was made in the state.^24^ For these reasons, in February 2020 the HSCRC announced the metric would no longer be part of the QBR program.

Although the first effort at including an ED boarding metric in HSCRC’s QBR program was short-lived, the inclusion of such a metric should be reconsidered. Several possible explanations exist for the lack of improvement in ED boarding despite previous inclusion of the ED-2b metric in Maryland’s QBR program. Most simply, shifting hospital operations and workflow is a difficult process that requires time. Second, given public notice of CMS’s proposed rule change, hospital executives had a diminished incentive to react to a quality metric that they perceived as transient. Lastly, the financial penalties tied to excessive ED-2b times may have simply been too small to matter. The solution to all these potential issues may be similar. A meaningful financial incentive tied to ED boarding metrics that is implemented on a long-term basis is highly likely to encourage hospital innovation to optimize patient access to emergency services.

Funding for the HSCRC Quality Pay-for-Performance programs comes from “at risk” global budget revenue. For rate year 2020, HSCRC allocated −2%/+2% of this revenue to QBR of which 50% was for “person and community engagement,” which included Hospital Consumer Assessment of Healthcare Providers and Systems survey domains and two ED wait-time metrics.^24^ While not explicitly partitioned, a rough estimate suggests that this placed less than a tenth of a percent of the global budget revenue at risk for ED wait time. Balanced against other quality measures, this incentive was arguably too small to prevent ED boarding. The HSCRC has the flexibility to pick and choose not just which measures to include but how heavily they are weighted.

The state’s regulatory authority for emergency services, MIEMSS, has also proposed strategies to reduce delays in emergency care. In November 2019 a Joint Chairmen’s Report directed MIEMSS to work with the HSCRC to provide a status update on various initiatives aiming to mitigate ED crowding.^11^ In addition to adding ED boarding measures to hospital quality reimbursement incentives, the report proposed that hospitals formulate action plans for improving efficiency, re-evaluating the use of yellow alerts for indicating diversionary status, identifying a standard for ambulance unloading time that would adapt to real-time ED crowding, and developing new models of EMS care delivery, such as mobile integrated health and community paramedicine. The use of yellow alerts should indeed be reevaluated and perhaps standardized at the state level rather than based on hospital policy, so that there is less variability in these alerts’ use. This revision could be implemented in parallel with the financial incentive previously discussed.

Addressing the underlying causes of shortages in staffed inpatient beds will support additional innovations and strategies to reduce ED boarding. Previous research suggests that one cause of inpatient bed shortages may be day-to-day variation in bed availability.^25^ This variation can occur due to elective surgeries being scheduled early in the week during times of higher ED demand, or fewer discharges occurring on the weekend due to decreased staffing. Guidelines or incentives could be considered for increased weekend staffing of personnel such as social workers, physical therapists, and case managers to improve weekend discharge efficiency. Notably, prior work has demonstrated that interventions aimed at smoothing surgical schedules and discharge planning improve ED throughput.^26^ While these administrative innovations can improve hospital flow in any reimbursement environment, they are particularly appealing under a global budget system. Financial incentives may induce hospitals to avoid using ED boarding to compensate for excess inpatient volumes, improving efficient patient flow, and use of hospital resources.

Research to better understand causal linkages between the current global budget system, shortages of inpatient hospital beds, and increases in ED boarding will inform the potential interventions discussed above. Further work uncovering these linkages is likely to have impacts even beyond improving emergency care in the state of Maryland. Maryland’s global budget model has garnered interest elsewhere in the country as a means of controlling healthcare costs. Thus, it is crucial to understand and improve on imbalanced incentives before implementation of similar models in other states. Under current policy structure, cost savings from global budgets need to be weighed against the potential of decreased patient access to emergency health services. However, while this research is ongoing, our recommendations would be that a financial incentive tied to ED crowding be reconsidered and yellow alerts be standardized at the state level.

## CONCLUSION

Maryland’s model of hospital financing has evolved over 40 years with a largely successful implementation of global budgeting, decreasing Medicare spending and meeting quality targets across several domains. However, evidence suggests that increased ED boarding and ambulance diversion have emerged as unanticipated consequences of the policy. This limits the ED’s ability to provide high-quality care for all patients and decreases access to care for vulnerable patient populations. These unintended consequences are likely to diminish the capacity to fulfill critical emergency care and safety net functions and may widen existing health disparities. Policymakers and hospitals alike should take actions to remedy the unintended consequences of the global budget.
